# Intramammary Infections in Heifers—Time of Onset and Associated Risk Factors

**DOI:** 10.3390/ani10061053

**Published:** 2020-06-18

**Authors:** Julia Nitz, Volker Krömker, Doris Klocke, Nicole Wente, Yanchao Zhang, Martin tho Seeth

**Affiliations:** 1 Department of Bioprocess Engineering, Microbiology, Faculty 2, University of Applied Sciences and Arts, 30453 Hannover, Germany; julia-nitz@web.de (J.N.); doris.klocke@hs-hannover.de (D.K.); nicole.wente@hs-hannover.de (N.W.); yanchao.zhang@hs-hannover.de (Y.Z.); 2 Department of Veterinary and Animal Sciences, Section Production, Nutrition and Health, Faculty of Health and Medical Sciences, University of Copenhagen, DK–1870 Frederiksberg C, Denmark; volker.kroemker@sund.ku.dk

**Keywords:** heifer, mastitis, risk factor, early lactation

## Abstract

**Simple Summary:**

Mastitis in dairy heifers during early lactation has global relevance and adverse impacts on milk production and milk quality. The aim of this study was to define the time-related period of intramammary infections and to relate this to risk factors for intramammary infections and subclinical mastitis by examining quarter milk samples of dairy heifers cytomicrobiologically. We worked out the important influence of early lactation on udder health and intramammary infections postpartum in dairy heifers. There is the possibility that udder quarters eliminate pathogens during early lactation, but there is also the danger that new infections manifest themselves. As related risk factors for new infections, the age at calving, udder edema, milk yield and somatic cell count after calving and detaching of milking cups during milking because of kicking off were determined. The prevention of new infections during the early lactation is an important purpose to ensure the future milk production and udder health in dairy heifers.

**Abstract:**

To reduce the negative effects of mastitis in dairy heifers in early lactation on the future milking performance, the aim of this study was to define the time-related period of intramammary infections and to relate this to risk factors at heifer and quarter level for intramammary infections and subclinical mastitis. In total, 279 German Holstein Frisian heifers in three farms in Northern and Eastern Germany were included in this study. Quarter milk samples for cytomicrobiological examination were collected 3 ± 1 days after calving and 17 ± 3 days after calving, and risk factors at heifer and quarter level associated with intramammary infections and clinical mastitis were recorded during the trial period. Data were analyzed using logistic regression procedures and odds ratios were calculated. Calving at older ages increased the odds of intramammary infections with non-*aureus* staphylococci (NAS) and coryneforms 17 ± 3 days after calving compared to heifers calving at a younger age. Detaching of milking cups during milking is a risk factor for new infections between day 3 ± 1 and 17 ± 3 postpartum. The milk yield after calving is associated with a decrease in intramammary infections with environmental pathogens and clinical mastitis. A high milk yield assists the development of udder edema with an increased risk of intramammary infections with NAS and coryneforms. An increased somatic cell count (SCC) after calving increased the odds of intramammary infections with contagious pathogens 17 ± 3 days postpartum. The early lactation has an important influence on udder health and intramammary infections postpartum in dairy heifers. Udder quarters eliminated pathogens during early lactation by 6.9% for cases in this study. New infections manifest themselves up until 17 ± 3 days postpartum, especially with *Corynebacterium* spp. and NAS. In total, 82.9% of the infected quarters showed new infections with another pathogen species 17 ± 3 days postpartum than 3 ± 1 days postpartum. In conclusion, the early lactation has an important influence on udder health and intramammary infections postpartum in heifers with the possibility that udder quarters eliminate pathogens, but also the danger that new infections manifest themselves. Thus, the prevention of new infections by minimizing the associated risk factors is of great importance.

## 1. Introduction

According to logic, heifers are not expected to develop intramammary infections (IMI) and subclinical mastitis (SCM) at first parturition because they were never previously milked. The daily milking routine entails risks regarding the transmission of contagious pathogens, and the milking vacuum has an adverse impact on the teat ends. Additionally, heifers’ udders are immature and seem to have less physical contact to the environment compared to cows [1].

Nevertheless, already in 1942, Schalm [2] indicated the existence of mastitis in heifers. In the 1980s, various authors took up the issue [3,4,5] and demonstrated the global incidence of infected udders of heifers at calving and early lactation to be high.

Reported prevalence of IMI and SCM in mammary quarters in heifers both pre- and postpartum varied widely between later studies. The prevalence of infected quarters at calving ranged from 18–80% [6,7,8]. SCM was found in 35% of early lactating heifers [9].

In 2009, Fox [1] indicated a median prevalence of IMI with non-*aureus* staphylococci (NAS) of 31.1% prior to calving and 27.9% after calving; thus, NAS were the most probable cause of IMI in heifers prepartum, as well as postpartum. Nonetheless, pathogens like *Staphylococcus (S.) aureus*, environmental streptococci, and coliforms also cause an IMI in heifers, both pre- and postpartum [1]. In former times, mastitis control programs in dairy cows focused on factors concerning the transmission of contagious mastitis pathogens, but not environmental pathogens [8]. Thus, the prevalence of contagious mastitis pathogens was reduced, and the prevalence of IMI with environmental pathogens increased in comparison [10]. However, the common mastitis prevention and control programs do not consider heifer mastitis [8,11].

IMI prior to calving and in the early lactation may interfere with the development of the mammary glands, the future milk production, udder health, and related culling hazards [12]. An IMI at calving also increases the risk of clinical mastitis (CM) within the first week after calving [13]. Edinger et al. [14] found that mastitis prior to calving and mastitis within the first week after calving increased the risk of further cases of mastitis and culling during the first 45 days of lactation. An elevated somatic cell count (SCC) in early lactation also negatively influences the test-day SCC during the entire first lactation [15]. De Vliegher et al. [16] stressed the negative effect of an elevated SCC postpartum on the milk production during heifers’ first lactation because of an impaired mammary function. Moreover, Coffey et al. [5] expect that heifers with a low first test day SCC produce more milk during the first lactation compared to those animals with elevated SCC in early lactation. Bludau et al. [11] detected a lower risk of either developing chronic mastitis or leaving the herd prematurely in heifers with no SCM during early lactation. Mastitis also decreases the reproductive performance during early lactation [17].

Until now, most studies considered the presence of IMI around calving, and only a few also determined SCC in quarter milk samples. Moreover, those studies using dairy herd improvement (DHI) samples in the early lactation only had composite samples available and IMI was not determined. Studies establishing both IMI and SCC in quarter milk samples are needed [18]. Furthermore, the exact moment at which the heifers’ udders are infected is not known. Thus, it is not possible to positively affect the rate of infections by correcting the related risk factors, although several risk factors for IMI and mastitis were already identified [8,11,13,19,20].

So far, in studies and in practice, the rate of mastitis in heifers detected in DHI tests was used to characterize the udder health of heifers in a herd, by calculating the percentage of heifers with an SCC > 100,000 cells/mL in the first test after calving among all heifers in the herd in their first test after calving. This makes herds comparable, but it is not possible to evaluate the precise moment of infection and the responsible risk factors. This is due to the fact that the timeframe between calving and the first day of testing differs between heifers as a consequence of the day of calving and the DHI sampling scheme. However, when high levels of mastitis in heifers are detected in the DHI tests, knowledge concerning times at which there is increased risk and related risk factors is necessary to prevent new infections.

In order to reduce the risk of mastitis in heifers having adverse impacts on future milk production and milk quality, it is necessary to identify the moment of infection and associated risk factors at heifer and quarter level. Thus, the aim of this study was to define the time-related period of infection of the udder of dairy heifers in early lactation and to relate this to risk factors for IMI and SCM at heifer level.

## 2. Materials and Methods 

### 2.1. Characteristics of Herds and Heifers

A total of 279 German Holstein Frisian heifers reared on three farms located in Northern and Eastern Germany were included in the study. The animals were selected according to their expected calving date between September 2017 and February 2018. All heifers that calved during the indicated timeframe were included in the study. In total, 20, 51, and 208 heifers on farms 1, 2, and 3 were enrolled in the trial. All of the herds participated in the local dairy herd improvement (DHI) association program (Lower Saxony or Saxony-Anhalt, respectively). The average herd size of the three study farms was 160, 221, and 784 lactating cows, and the average bulk milk SCC amounted to 150,000, 180,000, and 260,000 cells/mL during the trial period for farms 1, 2, and 3, respectively. Cows were milked two or three times per day in a herringbone or rotary parlor. All farms were equipped with a cubicle housing system.

The herds were selected from a database of the Microbiology group of the Faculty 2, Department of Bioprocess Engineering, University of Applied Sciences and Arts, Hannover, Germany, including farms which are regularly monitored for udder health. The herds were selected on the basis of a willingness to comply with the study protocol.

### 2.2. Quarter Milk Sample

The three farms were visited once a week between August 2017 and May 2018 and, in this time period, quarter milk samples and data on farm management practices and hygiene of animals and barns were collected.

Quarter milk samples of heifers were collected twice from all four quarters for SCC and microbiological diagnosis, 3 ± 1 days after calving and two weeks later at day 17 ± 3 after calving between September 2017 and March 2018, to define the postpartum IMI status. The exact time point of sampling the first sample after calving was when the period of colostrum was over, i.e., when the secretion had the characteristics of milk. All samples were aseptically collected by a veterinarian during the weekly visits or by the farmers after being instructed by the veterinarian in accordance with the guidelines of the German Veterinary Medical Association [21]. Accordingly, before collection, the apex of each teat was cleaned and disinfected with 70% ethanol and the first three streams of milk were discarded. Ten milliliters of milk was collected aseptically in sterile plastic tubes with boracic acid for preservation [22]. During the sampling process, disposable gloves were used and disinfected with 70% ethanol between heifers.

The heifers were observed by the herdsman and milkers for any signs of CM after parturition. CM is defined as the presence of typical inflammatory characteristics such as reddening, swelling, pain, a hot udder, or abnormal milk appearance like deviations in color or clotted milk and possibly systemic signs like fever and depression. Samples of quarters with a CM were collected by the farmers during the first 100 days of lactation in order to be able to investigate whether there is an association between infections up to the 17th day of lactation with the appearance of clinical mastitis until day 100. Quarters with functional failure at the time of the second quarter milk sample were no longer considered in the trial and were excluded from statistical analysis because of missing data.

The milk samples collected by the veterinarian were shipped in a cooling box (4–7 °C) to the laboratory (University of Applied Sciences and Arts, Faculty 2, Department of Bioprocess Engineering, Microbiology, Hannover, Germany) for further analysis at the day of sampling. Samples taken by the farmers were stored at 4–7 °C for a maximum of five days on the farms until transport to the laboratory for further analysis. 

### 2.3. Data Collection 

Several risk factors associated with CM at heifer and quarter level were gathered. Data relating to farm management were recorded using a questionnaire in an interview conducted with the farmers at the end of the trial period. The inspection of the heifers and barns regarding hygiene was carried out during the weekly farm visits in the period between eight weeks before the expected calving and about 17 ± 3 days after the real calving date. An overview of potential heifer and quarter level risk factors can be found in Table 1.

Potential risk factors at heifer level can be found in Table 1. The body condition score (BCS) of the heifers was determined eight weeks before the expected calving date and approximately 17 ± 3 days after calving. The visual scoring system developed by Edmonson et al. [23] was used for this purpose. This is based on a five-point scale using quarter point increments, ranging from 1 (emaciated) to 5 (obese).

The ease of calving was assessed by the farm personnel and recorded in the DHI record. The scale for ease of calving comprises the ranges 1 (unassisted calving/no difficulties), 2 (assistance required/low-grade difficulties), 3 (difficult birth), and 4 (Caesarean section).

The cleanliness of the heifers’ udders and thighs was determined eight weeks prior to the predicted calving and 17 ± 3 days after calving on a four-point scale by Schreiner and Ruegg [24]: completely free of dirt or having very little dirt (1), slightly dirty (2), mostly covered in dirt (3), and completely covered, caked-on dirt (4).

Ketosis after calving was ascertained by clinical signs and recorded by the farmers. The farmers differentiated between “clinical signs” or “no clinical signs” of the disease.

Occurring lameness was scored by the veterinarian during a period between eight weeks prepartum and 17 ± 3 days postpartum according to the locomotion scoring system of dairy cattle by Sprecher et al. [25].

Udder edema were assessed approximately 17 ± 3 days after calving. Edema was classified as “existing edema” and “no edema”, so that a distinction could be made between a long-lasting (existing 17 ± 3 days after calving) and a temporary (no edema 17 ± 3 days after calving) edema.

Potential risk factors at quarter level can be found in Table 1. The hygiene of the teat skin was scored visually before sampling for the absence or presence of dirt or manure. The teat end was cleaned with a swab, and a four-point scale was used to describe the amount of manure and litter sticking to the swab: “clean” (no manure, dirt, or dip), “dip present” (no manure or dirt), “small amount of dirt and manure present”, and “larger amount of dirt and manure present”. Cook and Reinemann [26] employed the scale after the preparation procedure prior to milking. In our study, it was used before cleaning the teat end and preparing the sampling approximately 17 ± 3 days after calving. Thus, an impression of heifers’ hygiene can be gained.

The teat end condition was recorded at day 17 ± 3 after calving. The evaluation was based on a four-point scoring system by Mein et al. [27]. This further developed the scoring system by Neijenhuis et al. [28] and is suitable for field evaluations. Teats with no teat end hyperkeratosis (N, no ring) are distinguished from teat ends showing callosity roughness. Teat end hyperkeratosis can be smooth or slightly rough (S, a raised ring with no roughness or only mild roughness and no keratin fronds), rough (R, a raised roughened ring with isolated old keratin fronds extending 1–3 mm from the orifice), or very rough (VR, a raised ring with rough old keratin fronds extending >4 mm from the orifice; the rim of the ring is rough and cracked giving the teat end a “flowered” appearance).

### 2.4. Laboratory Procedures 

The laboratory testing was performed at the microbiological laboratory, Faculty 2, Department of Bioprocess Engineering, University of Applied Sciences and Arts, Hannover, Germany. The milk samples were cultured and identified in accordance with the guidelines recommended by the German Veterinary Medical Association [21]. Ten microliters of each milk sample were inoculated onto an esculin blood agar plate (5% defibrinated sheep blood, Oxoid, Wesel, Germany). Plates were analyzed after 24 h and 48 h of aerobic incubation at 37 °C. The grown colonies were preliminarily differentiated by means of Gram staining, morphology and cell morphology, hemolysis patterns, and esculin hydrolysis.

Gram-positive, catalase positive cocci were categorized as NAS. Gram-positive, β-hemolyzing, catalase-producing cocci colonies were additionally tested for the clumping factor (Staph Plus Latex Kit, DiaMondiaL, Vienna, Austria) to verify *S. aureus* colonies from NAS. Esculin non-hydrolyzing streptococci were assigned to the Lancefield groups by means of the Strep Latex Kit (DiaMondiaL, Vienna, Austria). Esculin hydrolyzing streptococci were cultured on modified Rambach agar medium to check for ß-D-Galactosidase activity [29], positive colonies were differentiated as *S. uberis* and negative as *Enterococcus* spp.

Gram-positive irregular rods with Y-shaped cell configuration were identified as *Trueperella (T.) pyogenes*, if they were β-hemolytic and catalase negative. Gram-positive, non-hemolytic catalase positive irregular rods were categorized as coryneforms. Catalase positive Yeasts and *Prototheca* spp. were determined by microscopy. 

Gram-negative rods were differentiated by their ability to catabolize glucose under aerobic and anaerobic atmosphere (glucose supplemented oxidation-fermentation test medium, Merck, Darmstadt, Germany) and cytochrome C oxidase production (Bactident Oxidase, Merck, Darmstadt, Germany). Cytochrome C oxidase negative colonies fermenting glucose were cultured on Chromocult® Coliform Agar (Merck, Darmstadt, Germany) to distinguish *Escherichia (E.) coli* and other coliforms. Non-motile coliforms were additionally referred as *Klebsiella* spp. Gram-negative, cytochrome C oxidase positive bacteria which metabolized glucose oxidatively, were *Pseudomonas* spp. Following the guidelines of the German Veterinary Medical Association [21], the status of milk samples was defined. Thus, if at least one colony of the contagious pathogens *S. aureus, S. agalactiae, S. dysgalactiae*, or *T. pyogenes* was ascertained or more than 10 colonies of one of the other bacterial species, evidence of a correlation with an intramammary infection was given. A sample was considered contaminated if more than two different bacteria species were identified.

The SCC of the milk samples was ascertained by flow cytometry, using the SomaScope^TM^ Smart (Delta Instruments, Drachten, The Netherlands).

### 2.5. Definitions

Differentiating between persistent and new infection was based on cultural and biochemical identification of identical and non-identical pathogen species in the quarter milk samples 3 ± 1 days after calving and 17 ± 3 days after calving. 

Persistent infections were defined as IMI when the same pathogen species was detectable in the quarter milk samples 3 ± 1 days after calving and 17 ± 3 days after calving. 

New infections were defined as IMI 3 ± 1 days after calving and 17 ± 3 days after calving with different pathogen species in the same quarter, or IMI detectable 17 ± 3 days after calving in a quarter that was free of IMI 3 ± 1 days after calving.

Contagious pathogens get transmitted during milking from the infected quarter of one cow to another cow through the milker’s hands, liners, or milk [30]. 

Environmental pathogens get transmitted in stable areas and are stated in the cow’s environment [30]. 

### 2.6. Statistical Analysis

For analyzing the dataset, the program SPSS 25.0, Chicago IL, USA was used with the udder quarter as the statistical unit. Associations between IMI during the first 17 ± 3 days postpartum or mastitis during the first 100 days of lactation and risk factors (independent variables) were examined with generalized linear mixed models with logit link and binomial response (logistic regression) after pre-screening for variable selection in univariable analysis. 

The relationship between dependent and independent variables was tested first by means of the Student’s t-test for continuous measurements and with the χ^2^ test (likelihood ratio statistic) for proportions, with the exception of predictors in the same model, which indicate a correlation of *r* > 0.70 with one another. Then, independent variables associated with dependent variables at *p* < 0.10 in the Student’s test and χ^2^-test were submitted—when biologically plausible—to binary logistic regressions with IMI with contagious pathogens, IMI with environmental pathogens, IMI with NAS, or coryneforms and mastitis. Quarters with a missing value for one potential risk factor were not taken into account.

Using logistic regression procedures, the association between IMI during the first 17 ± 3 days postpartum or mastitis during the first 100 days of lactation and risk factors (independent variables) was examined, and the dependent binary dichotomous variables contained “new infection from day 3 ± 1 to day 17 ± 3 postpartum/no new infection from day 3 ± 1 to day 17 ± 3 postpartum”, “IMI with contagious pathogens/no IMI with contagious pathogens”, “IMI with environmental pathogens/no IMI with environmental pathogens”, “IMI with NAS or coryneforms/no IMI with NAS or coryneforms”, and “mastitis/no mastitis”. Herd, heifer within herd, and quarter within heifer were considered as random effects.

A backward stepwise procedure was used to select the final multivariable regression model. Potential risk factors were excluded one by one if *p* > 0.05.

Odds ratios (ORs) were calculated to describe the direction of the relationship between dependent and independent variables. ORs were determined with 95% confidence intervals (CI 95%) and a statistical significance at *p* ≤ 0.05. 

## 3. Results

### 3.1. Numbers of Heifers and Quarters

In total, 279 clinically healthy dairy heifers at the day of admission were included in the study on the three farms. Of these heifers, 51 were excluded before the study was completed. These animals left the study because of too few submitted samples, and eight quarters were no longer included in the study owing to functional failure at the time of the second quarter milk sample. Therefore, finally, 904 udder quarters were available for calculations. Numbers and proportions of heifers and quarters included in the analyses for assessing the risk factors are given in Table 2. 

### 3.2. Bacteriological Examination 

In total, 904 quarters were cytomicrobiologically examined 3 ± 1 days after calving and 17 ± 3 days after calving. The results are shown in Table 3. 

The percentages of quarters with no detected pathogen were 80.2% (*n* = 725) 3 ± 1 days after calving and 85.8% (*n* = 775) 17 ± 3 days after calving. Thus, 6.9% more quarters were non-infected 17 ± 3 days after calving compared to 3 ± 1 days after calving. In 19.8% (*n* = 179) of the quarters, IMI was detected 3 ± 1 days after calving, mostly isolated pathogens were NAS 4.8% (*n* = 43), and 4.7% (*n* = 42) were infected with *Pseudomonas* spp. On the other hand, 17 ± 3 days after calving, NAS 7.1% (*n* = 64), *Staphylococcus aureus* 2.1% (*n* = 19), and *Corynebacterium* spp. 1.4% (*n* = 13) were found in high percentages and, in total, 14.3% (*n* = 129) of all quarters were infected. 

### 3.3. Ratio of New Infections to Persistent Infections of the Samples with Pathogens Detected 17 ± 3 Days Postpartum

Table 4 splits the 129 samples of quarters with pathogens detected at 17 ± 3 days postpartum (Table 3) into samples showing new infections and samples showing persistent infections since 3 ± 1 days postpartum. Of these 129 quarters, 22 (17.1%) showed persistent infections, which means that the same pathogen species was detectable 3 ± 1 and 17 ± 3 days postpartum, seen especially in *Pseudomonas* spp. A total of 107 (82.9%) of the 129 infected quarters showed new infections, because there were no respective pathogen species detected 3 ± 1 days postpartum compared to 17 ± 3 days postpartum. Thus, 17 ± 3 days postpartum, 19 quarters were infected with *S. aureus*, whereby three of these quarters were already infected with *S. aureus* 3 ± 1 days postpartum, and 16 of these quarters were infected with another pathogen species or non-infected. NAS, *S. aureus*, and *Corynebacterium* spp. constituted a major proportion of the pathogen species 17 ± 3 days postpartum. The 107 new infected quarters 17 ± 3 days postpartum resulted from 83 quarters with no detected pathogen and 24 quarters with a detection of another pathogen species at day 3 ± 1 postpartum.

### 3.4. Risk Factors in Heifers and Quarters Associated with Intramammary Infections and Clinical Mastitis Postpartum

Results of final logistic regression models for the probability of a quarter developing an IMI or clinical mastitis are given in Table 5. The higher SCC at the first dairy herd improvement test after calving the higher the risk of having an IMI with contagious pathogens 17 ± 3 days postpartum (OR = 1.001). Heifers showing a persistency strong udder edema showed an increased risk of IMI with NAS and coryneforms three days postpartum, approximately 4.3 times greater (OR = 4.269) compared to heifers without udder edema. High milk yield recorded at the first improvement test after calving had a greater risk of IMI with NAS and coryneforms 3 ± 1 days postpartum (OR = 1.084), but a lower risk of IMI caused by environmental pathogens 17 ± 3 days postpartum (OR = 0.883) and clinical mastitis during the first 100 days of lactation (OR = 0.625). Heifers calving at older ages were more likely to have IMI with NAS and coryneforms 17 ± 3 days postpartum by about 25% each month than heifers calving at a younger age. Detaching of milking cups because of kicking off during milking was associated with multiplicity of new infections from day 3 postpartum to day 17 ± 3 postpartum by about 2.6 times (OR = 2.63). 

## 4. Discussion

This study focused on determining the period of the infection of the udder of dairy heifers in early lactation and aimed to relate this to risk factors at heifer level for IMI and SCM. Herd-specific risk factors were not taken into account. Identifying the moment of infection and risk factors associated with the infection of the udder is necessary for reducing the negative effects of mastitis in heifers on the future milking performance.

An infection of a mammary gland depends on exposure to microorganisms, udder defense mechanisms, and environmental risk factors [31] and, thus, can be influenced by external factors. Under certain circumstances, an invasion of the udder with pathogens can develop into a subclinical or clinical mastitis because of stressors. Previous studies showed that udders infected during the early lactation are at greater risk of negative performance concerning udder health, milk yield, or longevity [12].

For the present study, heifers from three dairy farms were examined. The farms were selected by coincidence and regarding willingness to participate in the study. Therefore, there was no representative selection. Nonetheless, the three farms are typical of Northern German farms. With regard to the number of heifers included in the study, this selection criterion seems sufficient to transfer the results to heifers from other farms.

We performed a cross-sectional study, where the unit of investigation was the heifer’s udder quarter. With an estimated prevalence of 0.2 and a precision of 0.05, assuming a sensitivity of 0.7 and a specificity of 0.9, a sample of 733 udder quarters was required. The date of the first sampling was defined as close as possible to the calving day, to map the status of infection at the time of this event. Sampling closer to calving than 3 ± 1 days postpartum would influence the results of examination because of the characteristics of the colostrum. Colostrum is characterized by a yellowish color and a viscous consistency. The duration of the period of colostrum differs between heifers. The exact time of sampling was chosen by observation, when the characteristics of colostrum were no longer maintained. However, the time frame was quite limited between 3 ± 1 days postpartum. The second sampling was set with an interval of 14 days from the first sampling. Therefore, the present IMI in the second sample can be separated from IMI in the first sample. Gröhn et al. [32] described a time interval of more than 14 days between two cases of CM as an indication for a new case. The use of this sampling scheme entails the risk that contaminations are misinterpreted as infections. Using multiple samplings might reduce this risk. However, infections caused by microorganisms that have a short duration of infection might be misinterpreted when using multiple samplings. According to Dohoo et al. [33], triplicate sampling provides only a modest gain in specificity and little or no gain in sensitivity compared to single sampling. Based on the reasons listed above, the implemented study design was chosen. The IMI status of heifers was determined in early lactation by bacteriological examinations and, thus, the time of infection was narrowed down. Up until now, the results of the first DHI record postpartum form the basis for the inflammation status. The date of the first DHI-record after calving depends on the day of calving and the scheme of sampling by the DHI. Hence, a distinction between infections until the period of colostrum and the time afterward is not possible. Our results show that 82.9% (Table 4) of current IMI 17 ± 3 days postpartum were non-existent 3 ± 1 days postpartum and, therefore, must be considered as new infections.

### Prevalence and Risk Factors for IMI

There was an association between an elevated SCC after calving and IMI with contagious pathogens 17 ± 3 days postpartum. In previous studies, Eberhart et al. [34] and Dohoo and Leslie [35] pointed out the relationship between an elevated SCC and an increased risk of IMI in cows. In particular, an IMI with contagious pathogens increased the risk of subclinical mastitis in early lactation [7,20]. Furthermore, the probability of CM increased in heifers with an increased initial SCC [36]. De Vliegher et al. [15] and Coffey et al. [5] figured out that heifers with low SCC after calving have a lower risk of SCM during the whole lactation period. De Vliegher et al. [15] compared heifers’ SCC records and found out that SCC increases postpartum and decreases from day five postpartum, with its lowest value at day 14. Thus, there appears to be no interference between the increased SCC 17 ± 3 days postpartum, which was shown in this study, and a physiological increase in the SCC postpartum.

The milk yield at the first DHI test may play an important role in the appearance of CM, as well as IMI with environmental pathogens and NAS and coryneforms. There seems to be an association between an increase in milk yield and a protection of the udder from IMI with environmental pathogens and CM. Nonetheless, the high yield seems to be accompanied by IMI with NAS and coryneforms. Previous studies described a higher milk yield in heifers and multiparous cows with an infection with NAS [7,20,37]. An explanation could be a protective effect of the IMI with NAS against IMI with other pathogens, according to Reference [7]. Furthermore, Piepers et al. [7] assumed that NAS-positive animals have a decreased incidence of CM and subsequently less loss of milk yield compared to animals without NAS infections. Piepers et al. [38] also presumed decreased CM cases during lactation in heifers with an NAS IMI during early lactation compared to those animals without NAS IMI. Furthermore, they described a difference in test day milk yield of 2.0 kg/day between infected and non-infected heifers with NAS. On the other hand, Compton et al. [20] suspected that cows with a higher milk yield might be more susceptible to IMI with NAS than those animals with a lower milk yield. Furthermore, Piccart et al. [39] discovered a lower quarter milk yield in quarters with NAS IMI compared to non-infected quarters, albeit during the acute phase of inflammation. According to these previous studies, there seems to be an association between an infection with NAS and coryneforms three days postpartum and the milk yield at the first DHI test after calving. Thus, either an infection is associated with an increase in the milk yield or an increased milk yield is predisposing for IMI.

In addition, the risks of infection with environmental pathogens 17 ± 3 days after calving and CM during the first 100 days of lactation appear to be lower in those animals with higher milk yield at the first DHI test. An explanation could be the increasing physical flushing effect, which reduces the number of pathogens and prevents a CM [20].

A prolonged udder edema is related to an increased risk of developing IMI with NAS and coryneforms three days after calving. Otherwise, Piepers et al. [8] identified a moderate to excessive udder edema as an important risk factor for IMI with contagious major pathogens in heifers. Moreover, Slettbakk et al. [40], Waage et al. [41], and Compton et al. [20] showed an association between udder edema and CM in heifers. A possible explanation is a defect in the local blood circulation with impairment of the immune response in the udder tissue. The inflammation, for its part, disturbs the circulation in the udder tissue and causes edema. Moreover, an increase in milk leakage caused by udder edema and, thus, an increased risk of CM is presumed [41]. Krömker et al. [13] assumed that heifers with udder edema are at greater risk of having open teat canals antepartum and open teat canals increase the risk of infections [6].

Heinrichs et al. [42] recognized a connection between udder edema around calving and diet-related factors, with Compton et al. [20] also showing elevated beta-hydroxybutyric acid (BHBA) concentrations and a high BCS loss to be risk factors for udder edema. In the present study, heifers with udder edema showed a greater BCS loss than those animals without edema.

Constable et al. [43] identified an increase in blood flow shortly before calving as the most important reason for udder edema. A higher milk yield at the start of lactation results in a higher risk of edema, whereas a longer time needed for vessels to adapt results in the edema lasting longer.

There seems to be an association between the age of calving and IMI with NAS and coryneforms 17 ± 3 days postpartum, just as Waage et al. [44] detected an association between an increase in age at first calving and an increased risk of mastitis. With increasing age, heifers show a higher BCS compared to younger animals at calving. These results correspond to another study which showed an association between a high BCS in cows before calving and increased risk of IMI after calving [31]. Herds with a lower BCS during the last month before calving had less incidence of mastitis [45]. Those heifers with a higher BCS before calving also show a greater loss of weight. Hillreiner et al. [46] described an increased BHBA concentration during negative energy balance as a possible reason for higher mastitis susceptibility during early lactation of dairy cows because of an innate immune function impairment in the udder.

It could be shown that detaching of milking cups because of kicking off with resulting sudden air leakage are associated with new infections between day 3 and 17 ± 3 postpartum. Furthermore, Thompson et al. [47] described the effect of sudden vacuum losses during milking as a factor for an increased risk of IMI. They subjected teats to large vacuum fluctuations during milking and high pathogen contamination. By venting the long milk tube from the milking machine, the vacuum dropped suddenly. As a result, the rate of IMI increased compared to the control quarters. Thompson et al. [47] explained this with the rapid airflow toward the teat end, triggered by a sudden vacuum loss.

A possible reason for teat cups being kicked off is the individual milking temperament of the heifers. This can be affected by habituating the heifers to the milking parlor and the milking routine. Kutzer et al. [48] trained heifers in the milking routine at least 10 days prepartum, and these heifers showed less stepping and kicking during the udder preparation phase after calving compared to untrained animals. Previous studies verified differences between the individual temperaments of dairy cows and their milking behavior in the parlor [49]. They categorized the animals’ behavior on the basis of steps, kicks, and leg movement; thus, these reactions can be described as individual milking temperament. Furthermore, the milking personnel has a great influence on the kicking off and loss of teat cups and milking clusters because of different cows’ behavior as a reaction to the type of treatment by the milking personnel [50].

A relationship between teat shape and teat diameter and the incidence of mastitis was described by Hickman [51]. He found a positive relationship between cylindrical teats and teats with a greater diameter and the incidence of mastitis. As shown in the present study, there is a relationship between udder edema and teat cup fall-offs and new infections. Edematous teats are cylindrical and have a great diameter; thus, they may be at greater risk of teat cup fall-offs while kicking, resulting in udder infections.

## 5. Conclusions

The udder health of heifers in a herd was characterized by the rate of cases of mastitis occurring in heifers at the DHI tests. This index is used in practice, as well as in other studies, to describe heifers’ udder health. It indicates the percentage of heifers in the herd with an SCC > 100,000 cells/mL at the first test after calving. However, the time frame between calving and the first day of testing differs between heifers. This makes herds comparable, but it does not allow evaluating the moment of infection and the responsible risk factors. Nonetheless, knowledge about times of increased risk and related risk factors is important when the occurrence of mastitis is high among heifers at DHI tests.

Every additional month of age at calving increases the risk of IMI with NAS and coryneforms 17 ± 3 days after calving, as well as the probability of developing a higher BCS before calving, associated with a higher BCS loss after calving and udder edema. The high milk yield after calving is associated with lower IMI with environmental pathogens and CM; thus, a higher milk yield is preferable. Nonetheless, a high milk yield is jointly responsible for the development of udder edema with an increased risk of IMI with NAS and coryneforms. To prevent new infections between day 3 and 17 ± 3 postpartum, efforts should be taken to avoid detaching of milking cups during milking. Further studies should point out causalities between risk factors and resulting IMI.

The present study showed the important influence of early lactation on udder health and IMI postpartum in heifers. There is the possibility that udder quarters eliminate pathogens during early lactation (this was proven in 6.9% of cases in this study). However, there is also the danger that new infections manifest themselves up until 17 ± 3 days postpartum, especially with *Corynebacterium* spp. and NAS. The consequences may be CM. As related risk factors for new infections, the age at calving, udder edema, milk yield, and somatic cell count after calving and detaching of milking cups during milking were determined. The origin of intramammary infections, which lead to increased cell counts in the first dairy herd improvement tests after calving, is so far prescribed to the period before calving. The control strategies, therefore, focus on minimizing risk factors prior to calving. However, this study clearly shows that a large proportion of infections occur after calving and, therefore, dairy farms need additional control strategies that relate to this period to prevent new infections occurring during early lactation to ensure the future milk production and udder health in dairy heifers.

## Figures and Tables

**Table 1:** Overview of heifer und quarter level risk factors potentially related to IMI ^1^ in heifers.

Risk Factor	Description/Classification	Recording Method	Breakdown Categories
Heifer level			
Individual access to pasture before or after calving	Whether or not heifers have access to pasture before or after calving	Interview	Yes versus no
Age at calving	Age at calving expressed in month	DHI ^2^ records	≤versus = months
Body condition before and after calving	Five-point scale, eight weeks before and 17 ± 3 days after calving	Visual	≤versus≥
Difference in body condition score	Difference before versus after calving	Visual	≤versus ≥ points
Ease of calving	Four-point scale: unassisted, assistance required, difficult birth, Caesar	DHI records	= 4 classes
Individual heifer housed with dry cows before calving	Whether or not heifers were housed with dry cows the eight weeks before calving	Interview	Yes versus no
Hygiene score heifer	Thigh and udder on four-point scale eight weeks before and 17 ± 3 days after calving	Visual	≤ versus ≥
Ketosis after calving	Whether or not the heifer suffered a ketosis after calving	Interview	Yes versus no
Lameness	Whether or not the heifer was lame during the trial period (score 2–5)	Visual	Yes versus no
Milk yield after calving	Test-day milk yield after calving	DHI records	
Detaching of milking cups because of kicking off	Whether or not the milking cups frequently became detached	Interview	Yes versus no
SCC ^3^ after calving	Test-day SCC after calving	DHI records	
Season of calving	Two seasons of calving: autumn (September–November), winter (December–February)	DHI records	Autumn versus winter
Udder edema	Whether or not udder edema exists more than 17 ± 3 days after calving	Visual and palpation	Yes versus no
Use of teat sealant	Internal or external teat sealants were used or not before calving	Interview	No versus internal versus external teat sealant
Quarter level			
Hygiene score teat skin	Teat apex on four-point scale 17 ± 3 days after calving	Visual	
Teat apex condition	No ring, smooth or slightly rough, rough, very rough 17 ± 3 days after calving	Visual and palpation	
Teat lesions	Whether or not the teat was damaged during the first 17 ± 3 days of lactation	Interview und visual	Yes versus no

^1^ Intramammary infections. ^2^ Dairy herd improvement. ^3^ Somatic cell count.

**Table 2:** Number and proportion of heifers and quarters for the risk factors included in the analyses.

Independent Variable	N (%) of Quarters: 904 Quarters
Heifer level	
Access to pasture before or after calving yes	536 (59.29)
Age at calving (months)	
23	80 (8.85)
24	291 (32.19)
25	234 (25.88)
26	145 (16.04)
27	66 (7.03)
28	52 (5.75)
29	12 (1.33)
30	8 (0.88)
31	8 (0.88)
32	4 (0.44)
Missing	4 (0.44)
Body condition before calving	
2.25	8 (0.88)
2.5	24 (2.65)
2.75	20 (2.21)
3	107 (11.84)
3.25	148 (16.37)
3.5	148 (16.37)
3.75	8 (0.88)
4	4 (0.44)
Missing	437 (48.34)
Body condition after calving	
2.25	8 (0.88)
2.5	83 (9.18)
2.75	222 (24.56)
3	296 (32.74)
3.25	204 (22.57)
3.5	47 (5.20)
3.75	8 (0.88)
4	0
Missing	36 (3.98)
Difference in body condition score	
0	115 (12.72)
0.25	51 (5.64)
−0.25	138 (15.27)
0.5	24 (2.65)
−0.5	79 (8.74)
0.75	12 (1.33)
−0.75	40 (4.42)
1	0
−1	8 (0.88)
Missing	437 (48.34)
Ease of calving	
1	748 (82.74)
2	116 (12.83)
3	32 (3.54)
Missing	8 (0.88)
Heifers housed with dry cows before calving (yes)	753 (83.30)
Hygiene score heifer before calving	
1	272
2	139
3	44
4	8
Missing	441
Hygiene score heifer after calving	
1	355
23	32596
4	44
Missing	84
Ketosis after calving (yes)	8 (0.88)
Lameness (yes)	44 (4.71)
Detaching of milking cups because of kicking off (yes)	28 (3.10)
Season of calving	
Winter	506 (55.97)
Autumn	398 (44.03)
Udder edema (yes)	262 (28.98)
Missing	355 (39.27)
Use of teat sealant (yes)	0
Quarter level	
Hygiene score teat skin	
1	331 (36.62)
2	136 (15.04)
3	53 (5.86)
4	18 (1.99)
Missing	366 (40.49)
Teat apex condition	
1	186 (20.58)
2	314 (34.73)
3	37 (4.09)
4	5 (0.55)
Missing	362 (40.04)
Teat lesions (yes)	0

**Table 3:** Pathogens isolated from quarter milk samples 3 ± 1 and 17 ± 3 days after calving.

Pathogen	Number (%) of Isolated Pathogens	
	3 ± 1 Days After Calving	17 ± 3 Days After Calving
*Staphylococcus aureus*	24 (2.7)	19 (2.1)
*Streptococcus uberis*	4 (0.4)	0
*Pseudomonas* spp.	42 (4.7)	7 (0.8)
NAS ^1^	43 (4.8)	64 (7.1)
*Corynebacterium* spp.	4 (0.4)	13 (1.4)
*Bacillus* spp.	2 (0.2)	2 (0.2)
*Trueperella pyogenes*	16 (1.8)	5 (0.6)
Coliform bacteria	34 (3.8)	7 (0.8)
C-Streptococci	2 (0.2)	4 (0.4)
*Enterococcus* spp.	1 (0.1)	2 (0.2)
*Klebsiella* spp.	1 (0.1)	0
*Escherichia coli*	1 (0.1)	0
G-Streptococci	0	1 (0.1)
Prototheca	0	1 (0.1)
Mixed	5 (0.6)	4 (0.4)
In total	179 (19.8)	129 (14.3)
No specific growth	725 (80.2)	775 (85.8)

^1^ Non-*aureus* staphylococci.

**Table 4:** Cause of infections existing at day 17 ± 3 postpartum ^1^.

	Infected Quarters 17 ± 3 Days After Calving (% ^2^)	Persistent ^3^ Infections (% ^4^)	New ^5^ Infections (% ^4^)
*Staphylococcus aureus*	19 (14.7)	3 (15.8)	16 (84.2)
*Pseudomonas* spp.	7 (5.4)	1 (14.3)	6 (85.7)
NAS ^6^	64 (49.6)	15 (23.4)	49 (76.6)
*Corynebacterium* spp.	13 (10.1)	0	13 (100)
*Bacillus* spp.	2 (0.2)	0	2 (100)
*Trueperella pyogenes*	5 (0.6)	2 (40.0)	3 (60.0)
Coliform bacteria	7 (0.8)	0	7 (100)
C-Streptococci	4 (0.4)	1 (25.0)	3 (75.0)
*Enterococcus* spp.	2 (0.2)	0	2 (100)
G-Streptococci	1 (0.1)	0	1 (100)
Prototheca	1 (0.1)	0	1(100)
More than 1 pathogen	4 (0.4)	0	4 (100)
In total	129 (100)	22 (17.1)	107 (82.9)
		129 (100)	

^1^ Differentiation based on cultural and biochemical identification of identical and non-identical pathogen species in the quarter milk samples 3 ± 1 days after calving and 17 ± 3 days after calving. ^2^ Proportion of one pathogen group of all infected quarters 17 ± 3 days postpartum. ^3^ Persistent infections are defined as intramammary infections with the same pathogen species detectable in the quarter milk samples 3 ± 1 days after calving and 17 ± 3 days after calving. ^4^ Proportion of persistent infections with respect to new infections refers to all the infected quarters 17 ± 3 days postpartum. ^5^ New infections are defined as intramammary infections 3 ± 1 days after calving and 17 ± 3 days after calving with different pathogen species in the same quarter, or intramammary infections detectable 17 ± 3 days after calving in a quarter, which was free of intramammary infections 3 ± 1 days after calving. ^6^ Non-*aureus* staphylococci.

**Table 5:** Final logistic regression models for the probability of a quarter to develop an IMI ^1^ or clinical mastitis, shown as the association between dependent and independent variables.

Dependent Variable	Independent Variable	Β ^2^	SE ^3^	OR ^4^	95% CI ^5^	*p*-Value
IMI with contagious pathogens 17 ± 3 days postpartum	SCC ^6^ after calving	0.001	0.000	1.001	1–1.002	0.004
IMI with NAS ^7^ and coryneforms 3 days postpartum	Udder edema	1.765	0.522	4.269	1.392–13.093	0.011
IMI with NAS and coryneforms 3 days postpartum	Milk yield after calving	0.063	0.029	1.084	1.012–1.162	0.021
IMI with NAS and coryneforms 17 ± 3 days postpartum	Age at calving	0.221	0.084	1.247	1.058–1.471	0.009
IMI with environmental pathogens 17 ± 3 days postpartum	Milk yield after calving	−0.088	0.039	0.883	0.793–0.983	0.023
Mastitis during the first 100 days of lactation	Milk yield after calving	−0.470	0.209	0.625	0.415–0.941	0.024
New infection between day 3 and 17 ± 3 postpartum	Detaching of milking cups because of kicking off	0.823	0.389	2.63	1.091–6.350	0.021

^1^ Intramammary infection. ^2^ Regression coefficient. ^3^ Standard error of the mean. ^4^ Odds ratio. ^5^ 95% confidence interval. ^6^ Somatic cell count. ^7^ Non-*aureus* staphylococci.
